# The Effectiveness and Outcomes of Culturally Adapted Cognitive Behavioral Therapy Across Common Mental Health Conditions: A Meta-Analysis

**DOI:** 10.3390/bs16030356

**Published:** 2026-03-02

**Authors:** Zahra Wakif, Vanessa Ip, Mahwish Ali Khan, Nuzhat Azim, Nivashi Arulventh, Haadiya Saleem, Spencer Yung, Reena Besa, Maheen Juweria, Arooj Shaukhat, Rabia Khan, Fatima Nadeem, Farooq Naeem

**Affiliations:** 1 Department of Psychology, York University, Toronto, ON M3J 1P3, Canada; 2 Institute of Medical Science, University of Toronto, Toronto, ON M5S 3K3, Canada; 3 Centre for Addiction and Mental Health, Toronto, ON M6J 1H4, Canada; 4 Department of Psychology, University of Nottingham, Semenyih 43500, Selangor, Malaysia; 5 Department of Psychology, University of Toronto, Toronto, ON M1C 1A4, Canada; 6 Daphne Cockwell School of Nursing, Toronto Metropolitan University, Toronto, ON M5B 1Z5, Canada; 7 Department of Psychology, Toronto Metropolitan University, Toronto, ON M5B 1Z5, Canada; 8 Misericordia Community Hospital, Edmonton, AB T5R 4H5, Canada; 9 Mind & Connect, Harlow CM19 4QX, UK; 10 Global University Systems, Toronto, ON M5G 1K2, Canada; 11 Pharma-Medical Science College of Canada, Toronto, ON M2J 4V8, Canada

**Keywords:** culturally adapted cognitive behavioral therapy, cognitive behavioral therapy, culture, minority, mental health, meta-analysis

## Abstract

Culturally adapted cognitive behavioral therapy (CaCBT) is increasingly used to reduce disparities in mental health outcomes among ethnoculturally diverse populations. Although CBT is a well-established evidence-based intervention, little is known about CaCBT’s effectiveness across diagnostic groups and global contexts. This meta-analysis synthesizes CaCBT efficacy for common mental health conditions. Using PRISMA guidelines, five electronic databases were used to search for RCTs reporting mental health variables for CaCBT. Funnel plots, Egger’s test, and the trim-and-fill method were used to evaluate publication bias. Hedges’ g was used to compute effect sizes, and heterogeneity was assessed through DerSimonian and Laird I^2^ statistics. Variations in populations, settings, and adaptation strategies were accounted for through random-effects models. Sixteen articles (*n* = 4787) met the inclusion criteria. CaCBT was associated with significant reductions in anxiety (g = −0.86, 95% CI [−1.66, −0.07], *p* = 0.032), somatic symptoms (g = −0.89, 95% CI [−1.61, −0.16], *p* = 0.016), and improved emotion regulation (g = 1.50, 95% CI [0.72, 2.28], *p* = 0.0002), though adjusted models reduced effects. For depression, PTSD, stress, and quality of life, pooled estimates favored CaCBT but did not reach statistical significance and were characterized by substantial heterogeneity. Significant heterogeneity was noted across studies, demonstrating diverse cultural contexts and intervention methods. CaCBT demonstrated significant benefits for anxiety, somatic symptoms, and emotional regulation across diverse groups. While depression and PTSD had varying outcomes, overall trends support this culturally responsive intervention’s efficacy. Further research on CaCBT, including understudied populations and standardized adaptation methods, could improve global mental health equity.

## 1. Introduction

Approximately half of the population will develop a mental health condition, such as depression and anxiety, at some point in their lives (McGrath et al., 2023). In addition to significantly reducing personal functioning and quality of life, mental health conditions also impose a substantial global burden on healthcare systems; the economic costs of mental health disorders are projected to meet or exceed six trillion US dollars by 2030 (Bloom et al., 2011). As such, improving access to and the effectiveness of mental healthcare interventions must remain a global priority. Among the most empirically supported psychological interventions is cognitive behavioral therapy (CBT), a structured, time-limited approach that targets maladaptive thought patterns and behaviors to alleviate psychological distress (Beck, 2021). CBT has demonstrated robust efficacy across a wide range of mental health conditions, making it a cornerstone of evidence-based psychological practice (Hofmann et al., 2012).

Since CBT is rooted in Western traditions, it is essential to culturally adapt it to enhance its efficacy and relevance when working with people from diverse non-Western backgrounds (Hays & Iwamasa, 2006; Scorzelli & Reinke-Scorzelli, 1994). Such adaptations involve modifying therapeutic content, delivery, and context to align with the cultural values, beliefs, and experiences of specific groups (Hall et al., 2019). Importantly, cultural adaptations play a pivotal role in addressing disparities in mental healthcare access and treatment outcomes, which are often exacerbated by cultural and linguistic barriers (Barrera et al., 2013).

Evidence suggests that variations in cultural beliefs, such as attributing mental health symptoms to supernatural causes or viewing stress as inevitable, shape how individuals perceive illness and engage with treatment (Rathod et al., 2010; W. Li et al., 2017). Culturally adapted CBT (CA-CBT) has become increasingly prevalent in recent years. Cultural adaptation involves a systematic process of modifying evidence-based treatments to consider language, context, and cultural factors that influence a client’s worldview, help-seeking behavior, and expression of distress (Hwang, 2006). Beyond simple translation, adaptation involves incorporating culturally specific idioms and metaphors and aligning therapeutic goals with cultural values, such as collectivism or spirituality (Bernal et al., 2009). Emerging research has therefore highlighted the potential of culturally adapted CBT (CA-CBT) for superior reductions in psychopathology outcomes compared to unadapted interventions (Hall et al., 2016; Escobar & Gorey, 2018). For instance, a study by Acarturk et al. (2019) involving treatment-resistant Turkish adolescents with depression and anxiety reported that ten sessions of culturally adapted transdiagnostic CBT led to significant reductions in anxiety and depression symptoms, with gains maintained at a two-month follow-up. Additionally, a foundational meta-analysis of 76 studies found that culturally adapted interventions resulted in overall improved attendance rates and symptom reduction (Griner & Smith, 2006).

To a similar extent, recent work has found that South Asian cultures stigmatize mental health conditions and reaching out for support more than Western cultures do (Javed et al., 2021; Mohsin et al., 2025). Mental health support has a negative connotation in some cultures, with the affected individual and their family being stigmatized if an individual needs mental health support (Karasz et al., 2019). Thus, cultural adaptations of therapy may be able to better integrate how different mental health is perceived across cultures and adapt therapy in a way that maintains individual autonomy and respect.

Despite the growing body of research in CA-CBT, the literature remains fragmented, with a lack of comprehensive synthesis of its effectiveness across common mental health conditions. While individual studies and narrative reviews have provided valuable insights, there is a pressing need for a meta-analysis to consolidate the evidence, evaluate the overall efficacy of CA-CBT, and identify factors that may influence its outcomes. No work, to our knowledge, has quantitatively assessed studies where participants received CaCBT for mental health outcomes. This study aims to address this gap as a meta-analysis of the effectiveness and outcomes of culturally adapted CBT across common mental health disorders. Ultimately, this synthesis is essential for translating research into equitable practice. By investigating the value of cultural adaptation, this study aims to provide the evidence necessary to ensure CBT interventions are effective and relevant for diverse populations.

## 2. Method

This study was reported in accordance with the Preferred Reporting Items for Systematic Reviews and Meta-Analyses (PRISMA) guidelines (Page et al., 2021), and followed the PRISMA-ScR 2020 Item Checklist (Supplementary File S1). The review protocol was registered on the International Prospective Register of Systematic Reviews (PROSPERO; ID: CRD42022339644, https://www.crd.york.ac.uk/prospero/display_record.php?ID=CRD42022339644) accessed on 25 November 2025, in accordance with PRISMA items 24a and 24b (registration and protocol).

### 2.1. Information Sources and Search Strategy

A medical librarian (RB) developed the initial search strategy in collaboration with the research team. The comprehensive strategy incorporated both free-text terms and controlled vocabulary (e.g., MeSH, EMTREE, and APA Thesaurus terms) to capture four core concepts: (i) culturally adapted cognitive behavioral therapy; (ii) mental health and addictions; (iii) ethnically, gender, and sexually diverse populations; and (iv) treatment outcomes and quality of life. The finalized strategy was translated and executed across the following databases in October 2022: MEDLINE, Embase, and PsycINFO via Ovid; the Cochrane Central Register of Controlled Trials (CENTRAL); and Web of Science–Core Collection. No limits or filters (e.g., language, year, study design) were applied to maximize search sensitivity.

In this review, culturally adapted cognitive behavioral therapy (CaCBT) was defined pragmatically, based on descriptions provided in primary studies. Studies were eligible if authors explicitly stated that the CBT intervention had been adapted to align with the cultural, linguistic, religious, or contextual characteristics of the target population. Eligible adaptations included modifications to language delivery, use of culturally relevant examples, incorporation of culturally salient belief systems and values, adjustments to treatment structure or delivery format, or adaptation of therapeutic materials. Adaptations were not required to be manualized, theory-driven, or standardized across studies; rather, intentionality was inferred from authors’ explicit characterization of the intervention as culturally adapted.

This flexible, author-defined operationalization was adopted to capture the breadth of CaCBT implementations in the literature but may have contributed to between-study heterogeneity due to variability in the type, depth, and theoretical grounding of cultural adaptations.

### 2.2. Eligibility Criteria

Inclusion criteria were as follows: (i) randomized controlled trials (RCTs); (ii) adults (>18 years); (iii) culturally adapted cognitive behavioral therapy (CaCBT) interventions with any mental health condition outcomes; and (iv) articles in English. Exclusion criteria were as follows: (i) not exclusively RCTs; (ii) not a CaCBT intervention; (iii) reviews, meta-analyses, or protocols and other documents that do not report findings; (iv) self-help, guided self-help, web-based programs, apps, and digital formats; and (v) articles not reporting mental health outcomes.

### 2.3. Screening Procedure

Once the search results were imported into Covidence (2025), duplicates identified by the software were automatically excluded. The first phase was the title and abstract phase, where each research assistant was trained in screening by the research lead (ZW) to ensure reliability. The second phase was the full-text screening, where researchers reviewed the full text of the articles, voted on their inclusion or exclusion, and provided a reason for their decision, which helped reduce the risk of bias. The research lead (ZW) resolved any conflicts throughout the stages in consultation with FN, and the selected studies met the inclusion criteria, proceeding to the extraction phase.

Titles/abstracts and full texts were independently screened by two reviewers to either “include” or “exclude”, with disagreements resolved by consensus (or a third reviewer).

### 2.4. Data Extraction Process

Co-authors extracted data for the systematic and meta-analytic review. Each article was based on the Cochrane Handbook (T. Li et al., 2023). Extraction training was provided by the research lead (ZW), with supervision provided by FN. The extracted data included sample population, study design and setting, methods, risk of bias, description of intervention and control groups, outcome measures, process variables, and results. Outcomes were grouped into broader domains when studies assessed closely related constructs using different but conceptually aligned measures. PTSD outcomes were analyzed separately as a distinct diagnostic trauma-related construct with specific symptom profiles and validated disorder-specific measures. In contrast, stress and emotional distress outcomes were conceptualized as non-diagnostic indicators of general psychological distress, reflecting stress burden or symptom severity rather than formal psychiatric diagnoses. These outcomes were grouped to capture a shared underlying construct of dimensional distress, consistent with prior psychotherapy meta-analyses synthesizing subthreshold or transdiagnostic symptom measures. For example, stress and emotional distress outcomes were analyzed due to their conceptual overlap and the use of related or composite symptom measures across included studies. To preserve the independence of observations, each study contributed only one effect size to any single pooled analysis.

Data extraction was conducted by a single reviewer using a standardized extraction form on Covidence. To minimize errors, extraction procedures were predefined, and data were cross-checked against original study reports where ambiguities arose.

In trials with multiple intervention arms, only comparisons relevant to the review question were included. For example, in Alegría et al. (2014), only the face-to-face culturally adapted CBT arm was compared with usual care. The telephone-delivered CBT arm was excluded to maintain consistency with the review’s focus on therapist-delivered, non-digital CaCBT interventions and to avoid non-independence of comparisons. Each study, therefore, contributed only one intervention-control comparison to any pooled analysis.

### 2.5. Data Analysis

A random-effects model was used to examine outcomes, allowing for variation in observed intervention effects due to fundamental intervention differences and sampling variability. This model was chosen as it accounts for heterogeneity across studies. We performed statistical analyses using R Statistical Software (v4.4.1; R Core Team, 2024) and the metafor package (v4.6-0; Viechtbauer, 2010). Study characteristics, including sample population and intervention details, are summarized in Table 1.

Sensitivity analyses based on risk of bias were not conducted. All eligible studies were retained in the quantitative synthesis to preserve statistical power and avoid selective exclusion in a relatively small and heterogeneous evidence base. Instead, risk of bias was assessed systematically and incorporated into the interpretation of findings, with methodological limitations explicitly described at the study level. As such, pooled effect estimates should be interpreted with caution, particularly where results were driven by studies with a higher risk of bias.

Included studies assessed mood-related outcomes using different validated instruments; standardized mean differences (SMDs) were used to enable quantitative synthesis across scales. Although standardized mean differences assume measurement equivalence across scales, differences in scale content, time frame, and sensitivity may introduce non-equivalence; accordingly, only conceptually aligned outcomes were pooled, and effect sizes were interpreted as relative rather than absolute effects.

Given the expected conceptual and contextual variability across populations, interventions, and outcome measures, pooled estimates are particularly problematic where heterogeneity is very high. These were interpreted as descriptive summaries of overall patterns rather than precise estimates of a single underlying treatment effect.

Primary analyses included the effectiveness of CaCBT on outcomes of depression, anxiety, stress, and post-traumatic stress disorder. Other psychosocial outcomes assessed were somatic symptoms, emotion regulation, and quality of life.

Forest plots were explicitly employed to illustrate the effect sizes of individual studies and the overall pooled effect, providing a visual assessment of the consistency of the results across studies. To evaluate heterogeneity among the included studies, we ran the DerSimonian and Laird I^2^ statistics. This statistic quantifies the percentage of variation across studies due to heterogeneity rather than chance (Higgins et al., 2003). This method supports the random-effects model, accommodating variability in study outcomes due to differences in study populations, interventions, and methodologies.

Funnel plots were generated to visualize the distribution of study effect sizes and standard errors. These analyses help determine the influence of any single study on the pooled effect size, confirming the stability and reliability of the findings. We utilized Egger’s test to assess publication bias, which detects asymmetry in funnel plots, a graphical representation used to identify potential bias in the meta-analysis results. When publication bias was detected, indicating that smaller studies with non-significant results might be missing, we applied the trim-and-fill method to estimate and adjust the summary effect size (Duval & Tweedie, 2000). This method quantifies publication bias by imputing potential missing studies and recalculating the effect size, including those missing studies.

Study quality was assessed independently by ZW, VI, NA, NA, MJ, AS, and RK using the Cochrane Risk of Bias Tool (Sterne et al., 2019) embedded in Covidence (2025), which evaluates six domains: random sequence generation (randomization), allocation concealment, blinding of participants and personnel, incomplete outcome data (attrition), selective outcome reporting, and other sources of bias (see Table 2). Study-level bias was rated as high if any individual domain scored high or if there were two or more unclear fields.

## 3. Results

**Figure 1:**
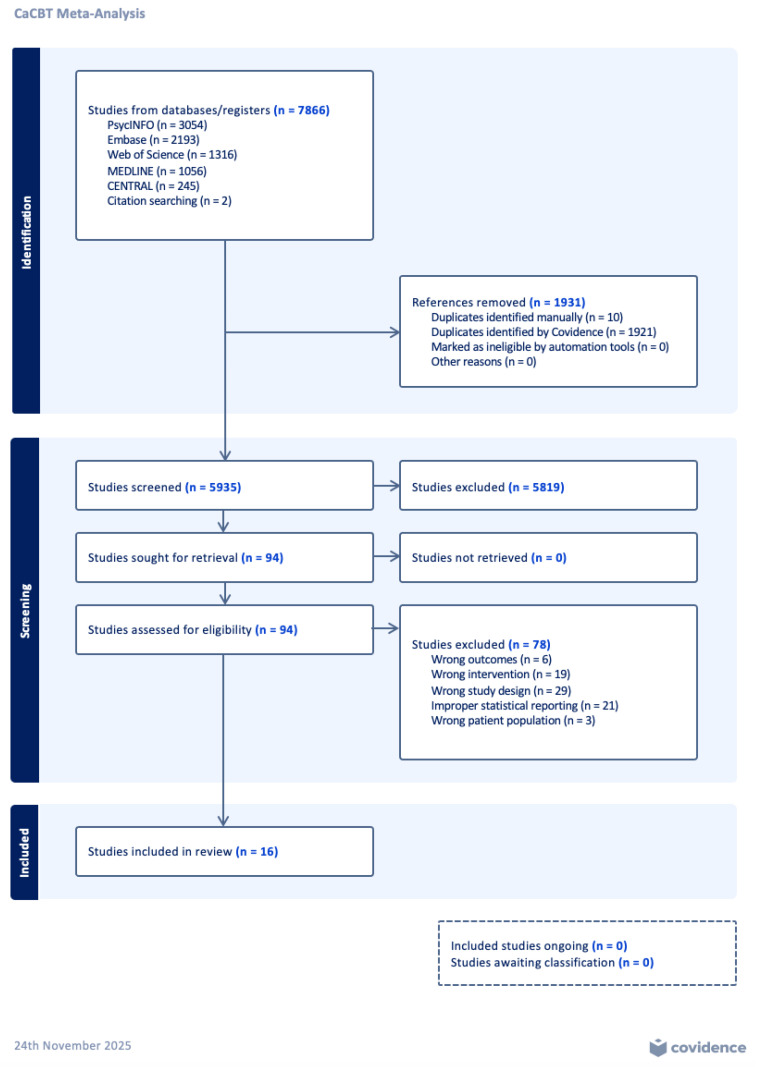
PRISMA flow diagram, generated through Covidence.

Our comprehensive search of the literature resulted in 37 studies that met our inclusion/exclusion criteria. During further full-text screening, 21 articles were removed due to improper statistical reporting. Thus, the final number of articles included is 16 (Figure 1). A total of 2465 participants were in the intervention group and 2322 in the control group, bringing the sample size to *n* = 4787. The mean age across the studies included ranged from 28.2 to 70.5 years, with reported standard deviations ranging from 6.1 to 14.28. Several studies reported mean age without an accompanying standard deviation, contributing to variation in the completeness of demographic data. Inclusion criteria for participant age generally fell between 16 and 65 years, with one study focused exclusively on older adults aged 60 years and above. The majority of study interventions were culturally adapted forms of CBT delivered in individual, group, telephone-based, brief, or lay-delivered formats (*n* = 14). Additional intervention categories, with some trials classified under multiple types, included trauma-focused CBT (*n* = 1) and CBT for adherence and depression (CBT-AD) (*n* = 1). With respect to comparison conditions, most studies used a treatment-as-usual (TAU) or routine-care control group (*n* = 7), followed by waitlist controls (*n* = 5) and active comparator interventions, such as applied muscle relaxation, standard CBT, or befriending therapy (*n* = 4). Intervention length ranged from 6 to 14 sessions across studies, with 12-session protocols being the most common (*n* = 7). Session duration was not consistently reported; most studies did not specify the length (in minutes) of each session. Where described generally, culturally adapted CBT sessions followed standard clinical practice and were typically delivered in approximately 45 to 90 min formats, though explicit durations were rarely provided. Several outcome-specific analyses were based on a limited number of studies (k = 3–5); therefore, pooled estimates for these outcomes should be interpreted as preliminary and exploratory rather than as stable effect estimates. For outcomes that did not reach statistical significance, results are described descriptively without implying trends or directional effects beyond the reported estimates. For all figures, studies are referenced with only the first author, followed by the year of publication.

Figure 1 demonstrates the PRISMA flow diagram, which outlines the study selection process used in this meta-analysis. A total of 7866 studies were identified in our initial search, with 1931 duplicates removed and uploaded to Covidence (2025) (Figure 1). After article screening, we excluded 5819 studies, leaving 94 full-text articles for assessment of eligibility. Upon full-text review and data extraction, 78 studies were excluded due to ineligible outcomes (*n* = 6), interventions (*n* = 19), study design (*n* = 29), improper statistical reporting (*n* = 21), or population (*n* = 3). Of the 37 studies included in the final review, 21 were excluded from quantitative synthesis due to improper or incomplete statistical reporting after five unsuccessful attempts to contact the authors of each article.

### 3.1. Anxiety

For studies measuring anxiety, a total of 1224 individuals were enrolled, with 257 withdrawals, resulting in a final sample of 967 participants. Six studies (*n* = 6) implemented intent-to-treat (ITT) analyses to account for missing data (Husain et al., 2024; Jalal et al., 2020; Naeem et al., 2015; Shaw et al., 2019; Yuan et al., 2020; Zemestani et al., 2022). Two studies (*n* = 2) did not report any information on attrition (Hinton et al., 2005, 2011), and one study (*n* = 1) used a per-protocol (completer-only) approach, excluding three dropouts from the final analysis (Eskici et al., 2021). One study (*n* = 1) acknowledged that dropout data were collected but deemed them unreliable, stating that “analyses were not performed because the reliability of the recorded data was low” (Karadere et al., 2024). Although the authors did not statistically adjust for attrition, they identified this as a study limitation rather than a methodological flaw, suggesting that incomplete participation data were unlikely to bias results. After accounting for losses to follow-up, 633 participants in the intervention groups and 588 participants in the control groups were included in the sample analyzed across the 10 studies reporting post-treatment outcomes. The sample comprised 1089 female and 132 male participants at enrollment, with five studies (*n* = 5) focusing exclusively on women (e.g., postnatal or refugee populations) (Eskici et al., 2021; Hinton et al., 2011; Husain et al., 2024; Shaw et al., 2019; Zemestani et al., 2022). The average mean age across studies was 40.7 years (SD = 7.5), calculated from nine studies (*n* = 9) reporting numerical age data (Eskici et al., 2021; Hinton et al., 2005, 2011; Husain et al., 2024; Karadere et al., 2024; Naeem et al., 2015; Shaw et al., 2019; Yuan et al., 2020; Zemestani et al., 2022). One study (*n* = 1) reported a mean age (28.2) without standard deviation (Jalal et al., 2020). One study (*n* = 1) included a concurrent non-randomized group, which was excluded to preserve independence of comparisons; the corresponding author was contacted to obtain the randomized treatment (G1) and waitlist control (G2) data used in the analysis (Shaw et al., 2019). Participants across the included studies primarily represented refugee, immigrant, and minority populations from South Asian, Middle Eastern, African, Caribbean, East Asian, and Southeast Asian backgrounds. These included Pakistani, Indian, Bangladeshi, Afghan, Iraqi Kurdish, Syrian, Cambodian, Dominican, Puerto Rican, Indigenous South African (Sepedi), Turkish, Han Chinese, and Caribbean-Latina communities. Studies were conducted across a wide geographic range, including the United Kingdom, the United States, Turkey, Malaysia, South Africa, China, Pakistan, and Iraq, reflecting diverse cultural and sociopolitical contexts.

**Figure 2:**
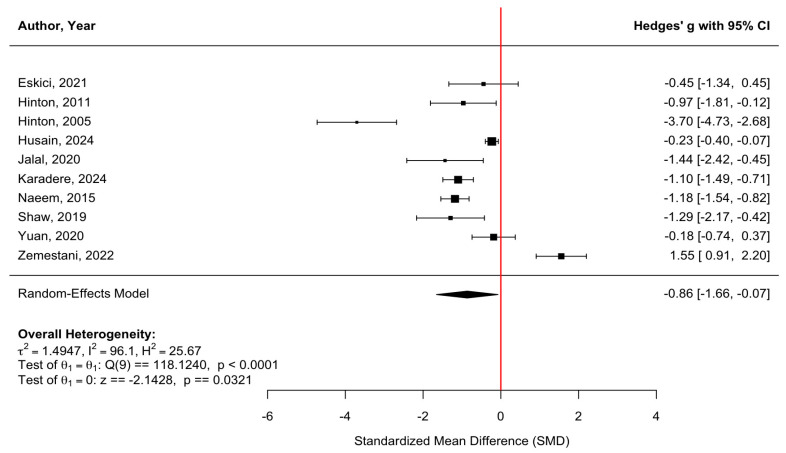
Forest plot of the effect of culturally adapted CBT on anxiety across diverse populations. Note. The following scales were used: **Hopkins Symptom Checklist–25 (HSCL-25)** (Eskici et al., 2021), **Hopkins Symptom Checklist-25—Anxiety Subscale (HSCL-25 [A])** (Shaw et al., 2019), **Symptom Checklist-90-Revised—Anxiety Subscale (SCL-90R [A])** (Hinton et al., 2011), **Anxiety Sensitivity Index (ASI)** (Hinton et al., 2005), **Generalized Anxiety Disorder Scale (GAD-7)** (Husain et al., 2024), **Hamilton Anxiety Rating Scale (HARS)** (Jalal et al., 2020), **Beck Anxiety Inventory (BAI)** (Karadere et al., 2024), **Hospital Anxiety and Depression Scale—Anxiety Subscale (HADS-A)** (Naeem et al., 2015), **Self-Rating Anxiety Scale (SAS)** (Yuan et al., 2020), and **Depression Anxiety Stress Scale—Anxiety Subscale (DASS-A)** (Zemestani et al., 2022).

**Figure 3:**
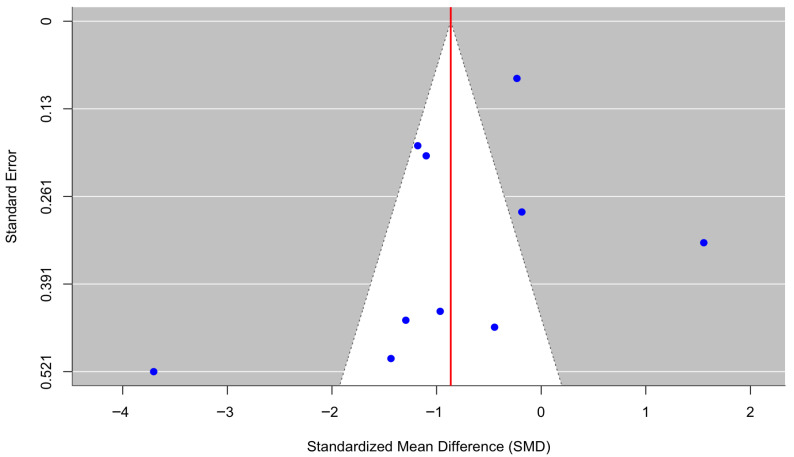
Funnel plot of the effect of culturally adapted CBT on anxiety across diverse populations using a random-effects model.

Figure 2 presents a forest plot of anxiety outcomes across 10 randomized controlled trials. Culturally adapted CBT produced a significant reduction in anxiety symptoms compared with control conditions, with a pooled effect size of g = −0.86 (95% CI [−1.66, −0.07], z = −2.14, *p* = 0.032). There was substantial heterogeneity across studies (τ^2^ = 1.49; τ = 1.22; I^2^ = 96.10%; H^2^ = 25.67), indicating considerable variability in effect sizes across populations and intervention formats (Q(9) = 118.12, *p* < 0.0001). Due to the very high heterogeneity (I^2^ = 96.10%), the pooled effect size should be interpreted with caution and is presented as a descriptive summary rather than a precise estimate of intervention efficacy. The funnel plot (Figure 3) did not indicate meaningful asymmetry, and Egger’s regression test was nonsignificant (z = −1.27, *p* = 0.204), suggesting no evidence of small-study effects or publication bias. Duval and Tweedie’s trim-and-fill procedure identified zero missing studies on the left and zero missing studies on the right (SEs = 2.0851 and 2.0440, respectively), and the adjusted pooled effect remained unchanged, reinforcing the robustness of the estimated treatment effect. For Karadere et al. (2024), the authors reported standard errors (SEs) rather than standard deviations; therefore, SDs were calculated using SD = SE × √n to ensure consistency of effect-size computations across studies included in the meta-analysis.

### 3.2. Depression

For studies measuring depression, a total of 1585 individuals were enrolled, with 300 withdrawals, resulting in a final sample of 1285 participants. Ten studies (*n* = 10) implemented intent-to-treat (ITT) analyses, and one study (*n* = 1) used a mixed-model (MAR) approach to account for missing data (Alegría et al., 2014; Dwight-Johnson et al., 2011; Husain et al., 2024; Hwang et al., 2015; Jalal et al., 2020; Kananian et al., 2020; Naeem et al., 2015; Simoni et al., 2013; Shaw et al., 2019; Yuan et al., 2020; Zemestani et al., 2022). One study (*n* = 1) did not report any information on attrition (Hinton et al., 2005), and one study (*n* = 11) used a per-protocol (completer-only) approach, excluding three dropouts from the final analysis (Eskici et al., 2021). One study (*n* = 1) acknowledged that dropout data were collected but were not analyzed, noting this as a limitation rather than a methodological flaw (Karadere et al., 2024). Although the authors did not statistically adjust for attrition, they stated that incomplete participation data were unlikely to bias results. After accounting for follow-up losses, 816 participants in the intervention groups and 766 participants in the control groups were included in the analyzed sample across the 14 studies reporting post-treatment outcomes. The sample comprised 1327 female and 255 male participants at enrollment, with four studies (*n* = 4) focusing exclusively on women (e.g., postnatal and refugee populations) (Eskici et al., 2021; Husain et al., 2024; Shaw et al., 2019; Zemestani et al., 2022). The average mean age across studies was 40.0 years (SD = 8.0), calculated from studies reporting numerical age data (Dwight-Johnson et al., 2011; Eskici et al., 2021; Hinton et al., 2005; Husain et al., 2024; Hwang et al., 2015; Kananian et al., 2020; Karadere et al., 2024; Naeem et al., 2015; Simoni et al., 2013; Yuan et al., 2020; Zemestani et al., 2022). Three studies (*n* = 3) reported mean age (35.4) but not standard deviation (Alegría et al., 2014; Jalal et al., 2020; Shaw et al., 2019). One study (*n* = 1) included a concurrent non-randomized group, which was excluded to preserve independence of comparisons; the corresponding author was contacted to obtain the randomized treatment (G1) and waitlist control (G2) data used in the analysis (Shaw et al., 2019). Participants across the included studies represented a wide range of immigrant, refugee, and minority groups from Latin American, Middle Eastern, South Asian, Southeast Asian, East Asian, African, and Turkish backgrounds. These culturally diverse samples included Latino populations from the U.S. mainland and Puerto Rico, Mexican and Mexican American communities, Syrian, Afghan, and Iraqi Kurdish refugees, Cambodian and South Asian women in the United Kingdom, Indigenous South Africans, Han Chinese older adults, and Chinese Americans. Studies were conducted across North America, Europe, the Middle East, South and Southeast Asia, and Africa, reflecting extensive cultural and geographic diversity.

**Figure 4:**
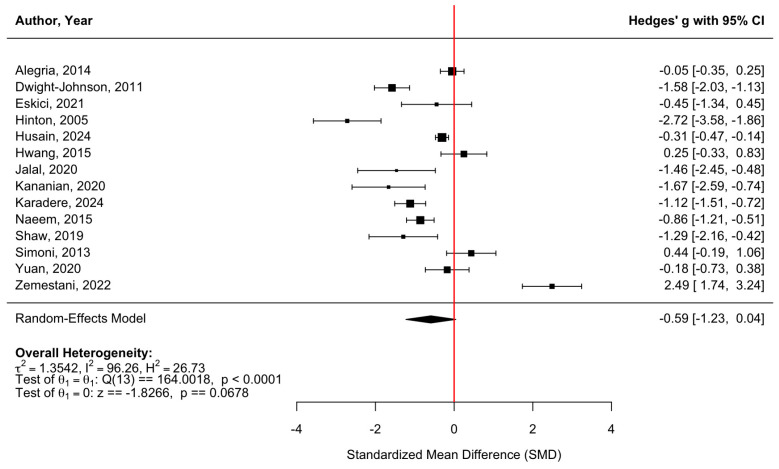
Forest plot of the effect of culturally adapted CBT on depression across diverse populations Note. The following scales were used: **Patient Health Questionnaire (PHQ-9)** (Alegría et al., 2014; Dwight-Johnson et al., 2011, Husain et al., 2024; Kananian et al., 2020), **Hopkins Symptom Checklist- 25 (HSCL-25)** (Eskici et al., 2021 **(HSCL-25-D)**; Shaw et al., 2019 **(HSCL-25-D)**), **Symptom Checklist-90 (SCL-90 A+D composite)** (Hinton et al., 2005), **Hamilton Depression Rating Scale (HDRS)** (Hwang et al., 2015; Naeem et al., 2015), **Beck’s Depression Inventory (BDI)** (Karadere et al., 2024; Jalal et al., 2020; **(BDI-II)** Simoni et al., 2013; **(BDI-IA)**), **Geriatric Depression Scale (GDS)** (Yuan et al., 2020) and **Depression, Anxiety, Stress Scale- Depression (DASS-D)** (Zemestani et al., 2022).

**Figure 5:**
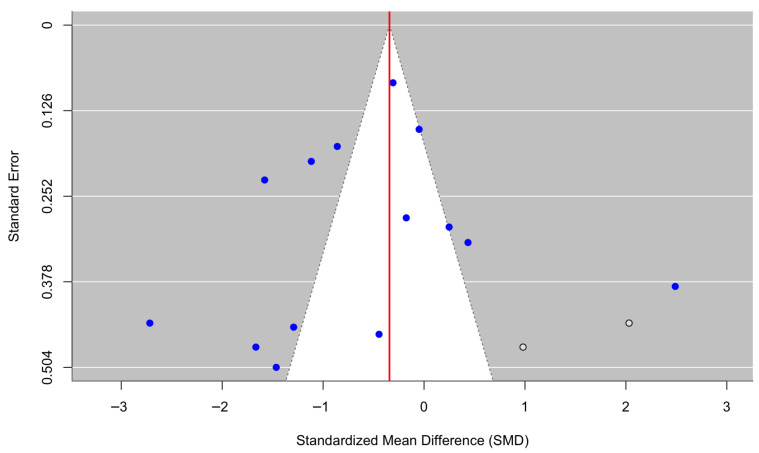
Adjusted funnel plot of the effect of culturally adapted CBT on depression across diverse populations using a random-effects model.

Figure 4 presents a forest plot of depression outcomes across 14 randomized controlled trials. Culturally adapted CBT was associated with a modest reduction in depressive symptoms compared with control conditions, with a pooled effect size of g = −0.59 (95% CI [−1.23, 0.04], z = −1.83, *p* = 0.0678). There was substantial heterogeneity among studies (τ^2^ = 1.35; τ = 1.16; I^2^ = 96.26%; H^2^ = 26.73), indicating considerable variability in effect sizes across populations, settings, and intervention formats (Q(13) = 164.00, *p* < 0.0001). Accordingly, the pooled estimate should not be interpreted as a stable or generalizable effect but rather as a summary of highly variable findings across culturally adapted interventions. The funnel plot (Figure 5) showed slight asymmetry; however, Egger’s regression test did not provide statistical evidence of small-study effects (z = −0.68, *p* = 0.495). Duval and Tweedie’s trim-and-fill procedure detected no missing studies on the left and two potentially missing studies on the right, yielding an adjusted model with k = 16. After imputation, the pooled effect size decreased to g = −0.34 (95% CI [−1.00, 0.32], *p* = 0.308). Neither the original nor the adjusted pooled estimates reached statistical significance, and the substantial between-study variability means the true effect remains uncertain. Consistent with other outcomes, for Karadere et al. (2024), reported standard errors (SEs) were converted to standard deviations (SDs) using SD = SE × √n to ensure comparability across studies.

### 3.3. Post-Traumatic Stress Disorder (PTSD) and/or Trauma

For studies measuring PTSD and/or trauma, 232 individuals were included, with 21 withdrawals, resulting in a final sample size of 211 participants. Four studies (*n* = 4) implemented intent-to-treat (ITT) analyses (Jalal et al., 2020; Kananian et al., 2020; Shaw et al., 2019; Zemestani et al., 2022). Three studies (*n* = 3) did not report any information on attrition (Hinton et al., 2005, 2009, 2011), and one study (*n* = 1) used a per-protocol (completer-only) approach, excluding three dropouts from the final analysis (Eskici et al., 2021). After accounting for losses to follow-up, 121 participants in the intervention groups and 108 participants in the control groups were included in the analyzed sample across the eight studies reporting post-treatment outcomes. The sample comprised 174 female and 55 male participants at enrollment, with four studies (*n* = 4) focusing exclusively on women (e.g., postnatal and refugee populations) (Eskici et al., 2021; Hinton et al., 2011; Shaw et al., 2019; Zemestani et al., 2022). The average mean age across studies was 40.1 years (SD = 6.6), calculated from studies reporting numerical age data (Eskici et al., 2021; Hinton et al., 2005, 2009, 2011; Kananian et al., 2020; Zemestani et al., 2022). Two studies (*n* = 2) reported mean age (30.6) but not standard deviation (Jalal et al., 2020; Shaw et al., 2019). One study (*n* = 1) included a concurrent non-randomized group, which was excluded to preserve independence of comparisons; the corresponding author was contacted to obtain the randomized treatment (G1) and waitlist control (G2) data used in the analysis (Shaw et al., 2019). Participants in these studies represented refugee, immigrant, and minority groups primarily affected by war, displacement, and sociopolitical violence. Samples included Syrian, Caribbean-Latina, Cambodian, Iraqi Kurdish, Afghan, and Indigenous South African populations. Many participants were trauma-exposed refugees with limited formal education, limited English proficiency, or experiences of forced migration. Studies were conducted across Turkey, the United States, South Africa, Germany, Malaysia, and Iraq, reflecting a broad range of cultural, linguistic, and geopolitical contexts.

**Figure 6:**
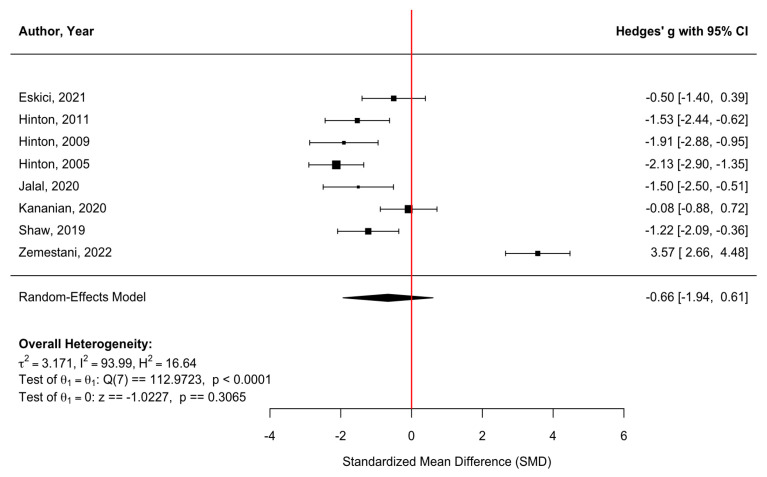
Forest plot of the effect of culturally adapted CBT on PTSD and/or trauma across diverse populations. Note. The following scales were used: **Harvard Trauma Questionnaire (HTQ)** (Eskici et al., 2021; Shaw et al., 2019), **Posttraumatic Stress Disorder Checklist (PCL)** (Hinton et al., 2011; Jalal et al., 2020); **(PCL-C)** (Kananian et al., 2020); **(PCL-5)**; (Zemestani et al., 2022); **(PCL-5)**; and **Clinician Administered PTSD** (Hinton et al., 2005, 2009).

**Figure 7:**
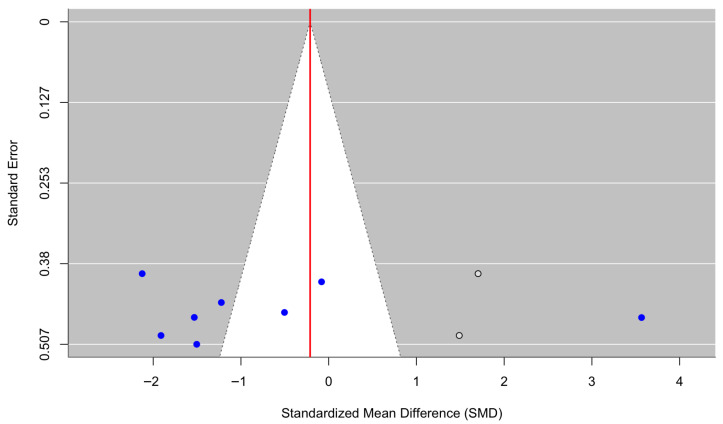
Adjusted funnel plot of the effect of culturally adapted CBT on PTSD and/or trauma across diverse populations using a random-effects model.

Figure 6 presents a forest plot of PTSD and trauma-related outcomes across eight randomized controlled trials. Culturally adapted CBT was associated with a nonsignificant reduction in PTSD symptoms compared with control conditions, with a pooled effect size of g = −0.66 (95% CI [−1.94, 0.61], z = −1.02, *p* = 0.3065). There was substantial heterogeneity across studies (τ^2^ = 3.17; τ = 1.78; I^2^ = 93.99%; H^2^ = 16.64), indicating considerable variability in treatment effects (Q(7) = 112.97, *p* < 0.0001). The funnel plot (Figure 7) did not suggest meaningful asymmetry, and Egger’s regression test was nonsignificant (z = 0.06, *p* = 0.954), providing no evidence of small-study effects or publication bias. Duval and Tweedie’s trim-and-fill procedure detected no missing studies on the left and two potentially missing studies on the right, yielding an adjusted model with k = 10. After imputation, the pooled effect decreased to g = −0.21 (95% CI [−1.38, 0.95], z = −0.36, *p* = 0.722). Although the adjusted estimate further attenuated the magnitude of the pooled effect, neither the original nor the adjusted pooled estimate was statistically significant. Given the very high heterogeneity (I^2^ = 93.99%), reflecting substantial variation in trauma exposure, cultural context, intervention focus, and outcome measurement, these results should be interpreted with caution and regarded as descriptive rather than confirmatory.

### 3.4. Stress and/or Emotional Distress

In this analysis, stress and emotional distress were conceptualized as non-diagnostic psychological distress and stress-related symptom severity, assessed using validated distress and symptom-burden measures. For studies measuring stress or emotional distress, a total of 257 individuals were enrolled, with five withdrawals, resulting in a final sample of 252 participants. Two studies (*n* = 2) implemented intent-to-treat (ITT) analyses to account for missing data (Kananian et al., 2020; Zemestani et al., 2022). Two studies (*n* = 2) did not report any information on attrition (Hinton et al., 2005, 2009). One study (*n* = 1) acknowledged that dropout data were collected but deemed unreliable, stating that “analyses were not performed because the reliability of the recorded data was low” (Karadere et al., 2024). Although the authors did not statistically adjust for attrition, they identified this as a study limitation rather than a methodological flaw, suggesting that incomplete participation data were unlikely to bias results. After accounting for losses to follow-up, 143 participants in the intervention groups and 114 participants in the control groups were included in the analyzed sample across the five studies reporting post-treatment outcomes. The sample comprised 177 female and 80 male participants at enrollment, with one study (*n* = 1) focusing exclusively on women (e.g., postnatal or refugee populations) (Zemestani et al., 2022). The average mean age across studies was 37.8 years (SD = 6.8), calculated from five studies (*n* = 5) reporting numerical age data (Hinton et al., 2005, 2009; Kananian et al., 2020; Karadere et al., 2024; Zemestani et al., 2022). Samples included Cambodian, Afghan, Turkish, and Iraqi Kurdish populations. Many participants were trauma-exposed refugees with limited formal education, limited language proficiency, and histories of forced migration, war-related trauma, or political violence. Studies were conducted across the United States, Germany, Turkey, and Iraq, reflecting a broad range of cultural, linguistic, and geopolitical contexts.

**Figure 8:**
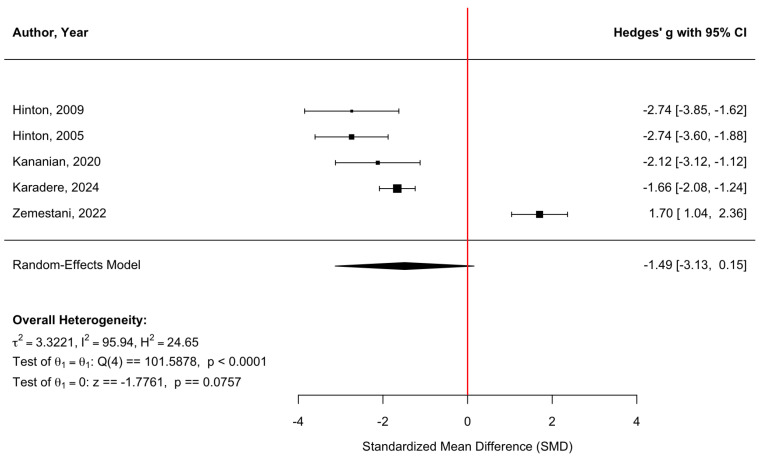
Forest plot of the effect of culturally adapted CBT on stress and/or emotional distress across diverse populations. Note. The following scales were used: the **Orthostatic Panic Attack Severity Scale (O-PASS)** (Hinton et al., 2005, 2009), the **General Health Questionnaire-28 (GHQ-28)** (Kananian et al., 2020), the **Yale–Brown Obsessive–Compulsive Scale (Y-BOCS)** (Karadere et al., 2024), and the **Depression Anxiety Stress Scale—Stress subscale (DASS-S)** (Zemestani et al., 2022).

**Figure 9:**
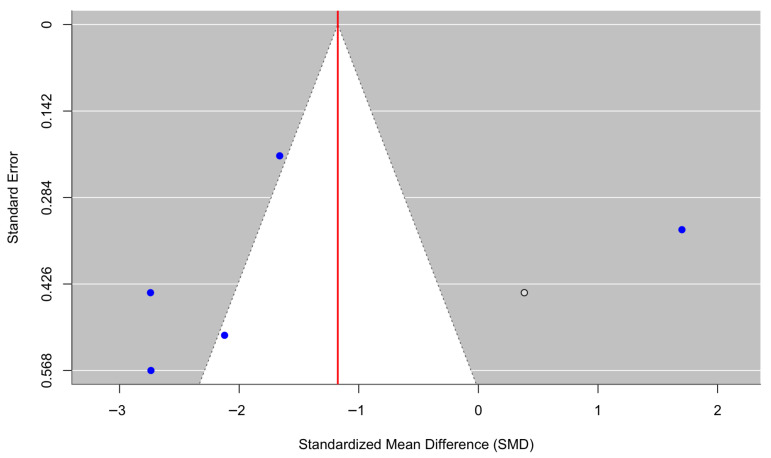
Adjusted funnel plot of the effect of culturally adapted CBT on stress and/or emotional distress across diverse populations using a random-effects model.

Figure 8 presents a forest plot of stress and emotional-distress outcomes across five randomized controlled trials. Culturally adapted CBT was associated with a nonsignificant reduction in stress symptoms compared with control conditions, with a pooled effect size of g = −1.49 (95% CI [−3.13, 0.15], z = −1.78, *p* = 0.0757). Heterogeneity was substantial (τ^2^ = 3.32; τ = 1.82; I^2^ = 95.94%; H^2^ = 24.65), indicating marked variability in effect sizes across populations, settings, and intervention approaches (Q(4) = 101.59, *p* < 0.0001). In line with other outcomes displaying high heterogeneity (I^2^ = 95.94%), reflecting differences in non-diagnostic distress constructs and measurement approaches, the combined effect should be interpreted carefully and not regarded as indicating a consistent treatment effect. The funnel plot (Figure 9) did not show statistically significant evidence of publication bias, and Egger’s regression test was nonsignificant (z = −0.91, *p* = 0.362), suggesting no detectable small-study effects. Duval and Tweedie’s trim-and-fill procedure estimated one potentially missing study on the right side, yielding an adjusted model with k = 6. After imputation, the pooled effect size attenuated to g = −1.18 (95% CI [−2.65, 0.29], z = −1.57, *p* = 0.117), reflecting a smaller and nonsignificant effect. Neither the original nor the adjusted pooled estimates reached statistical significance. As with other outcomes, for Karadere et al. (2024), standard deviations (SDs) were derived from reported standard errors (SEs) using SD = SE × √n to ensure consistency across studies included in the meta-analysis.

### 3.5. Somatic Symptoms

For studies measuring somatic symptoms, a total of 181 individuals were enrolled, with 38 withdrawals, resulting in a final sample of 143 participants. All three studies (*n* = 3) implemented intent-to-treat (ITT) analyses to account for missing data (Jalal et al., 2020; Kananian et al., 2020; Naeem et al., 2015). After accounting for losses to follow-up, 91 participants in the intervention groups and 90 participants in the control groups were included in the analyzed sample across the three studies reporting post-treatment outcomes. The sample comprised 97 female and 84 male participants at enrollment. The average mean age across studies was 39.5 years (SD = 7.4), calculated from two studies (*n* = 2) reporting numerical age data (Kananian et al., 2020; Naeem et al., 2015). One study (*n* = 1) reported mean age (28.2) without standard deviation (Jalal et al., 2020). The studies included in this subgroup primarily involved culturally and linguistically diverse populations from African, Middle Eastern, and South Asian backgrounds. These samples included Indigenous South Africans from the Sepedi cultural group, Afghan refugees representing Pashtun, Tajik, and Hazara ethnicities, and Urdu-speaking Pakistani adults. Research was conducted across South Africa (Limpopo Province), Germany (Frankfurt am Main), and Pakistan (Lahore), reflecting a mix of refugee, immigrant, and local minority communities within varied sociopolitical and healthcare contexts.

**Figure 10:**
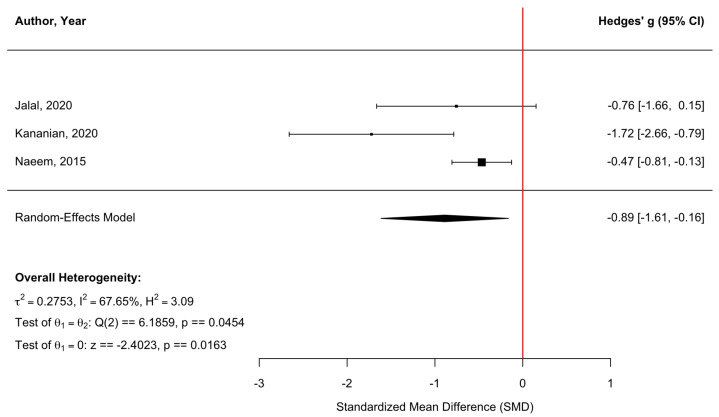
Forest plot of the effect of culturally adapted CBT on somatic symptoms across diverse populations. Note. The following somatic symptom scales were used: **SCL-90-R Somatization Subscale** (Jalal et al., 2020), **Somatic Symptom Scale-8 (SSS-8**) (Kananian et al., 2020), and **Patient Health Questionnaire-15 (PHQ-15)** (Naeem et al., 2015).

**Figure 11:**
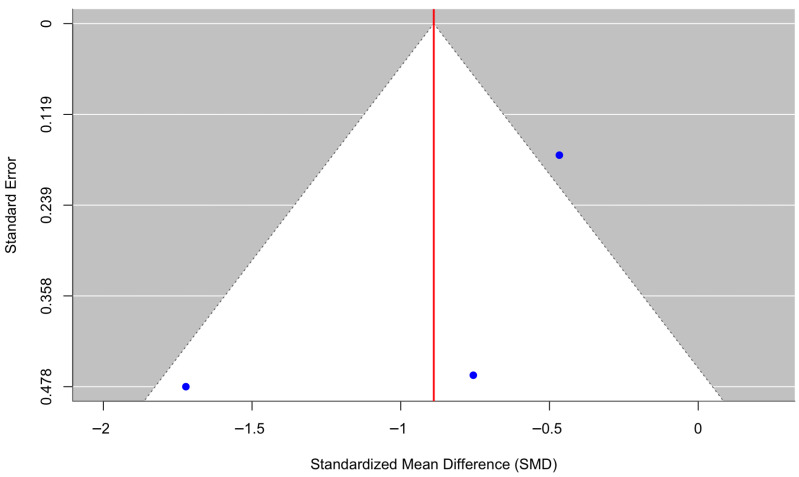
Adjusted funnel plot of the effect of culturally adapted CBT on somatic symptoms across diverse populations using a random-effects model.

Figure 10 presents a forest plot of three randomized controlled trials evaluating somatic symptom outcomes. Culturally adapted CBT demonstrated a significant reduction in somatic symptoms compared with control conditions, yielding a pooled effect size of g = −0.89 (95% CI [−1.61, −0.16], z = −2.40, *p* = 0.0163). Heterogeneity was moderate (τ^2^ = 0.28, τ = 0.52, I^2^ = 67.65%, H^2^ = 3.09), indicating variability across studies (Q(2) = 6.19, *p* = 0.0454). The funnel plot (Figure 11) showed no statistically significant evidence of small-study effects. Egger’s regression test was nonsignificant (z = −1.17, *p* = 0.241), and trim-and-fill analyses estimated no missing studies on either the left or right side. As a result, the pooled effect size remained unchanged after adjustment (g = −0.89, 95% CI [−1.61, −0.16], *p* = 0.0163), suggesting that publication bias is unlikely to have influenced the results. However, it should be noted that due to the small number of included studies, their findings should be interpreted cautiously.

### 3.6. Quality of Life

For studies measuring quality of life, a total of 1161 individuals were enrolled, with 245 withdrawals, resulting in a final sample of 916 participants. All six studies (*n* = 6) implemented intent-to-treat (ITT) analyses to account for missing data (Alegría et al., 2014; Husain et al., 2024; Kananian et al., 2020; Naeem et al., 2015; Yuan et al., 2020; Zemestani et al., 2022). After accounting for losses to follow-up, 583 participants in the intervention groups and 578 participants in the control groups were included in the analyzed sample across the six studies reporting post-treatment outcomes. The sample comprised 1022 female and 139 male participants at enrollment, with two studies (*n* = 2) focusing exclusively on women (e.g., postnatal or refugee populations) (Husain et al., 2024; Zemestani et al., 2022). The average mean age across studies was 37.7 years (SD = 6.2), calculated from five studies (*n* = 5) reporting numerical age data (Husain et al., 2024; Kananian et al., 2020; Naeem et al., 2015; Yuan et al., 2020; Zemestani et al., 2022). One study (*n* = 1) reported mean age (45) without standard deviation (Alegría et al., 2014). Participants across the included studies primarily represented refugee, immigrant, and minority populations from South Asian, Middle Eastern, East Asian, and Latin American backgrounds. These included Latino adults from the United States and Puerto Rico (White Latino, Black/dark-skinned Latino, mixed-heritage, and unspecified Latino), British South Asian women of Pakistani, Indian, Bangladeshi, and other South Asian origins, Afghan refugees from Pashtun, Tajik, and Hazara ethnic groups, Urdu-speaking Pakistani adults, Han Chinese older adults from rural Sichuan, and Iraqi Kurdish women exposed to war-related trauma. Studies were conducted across a wide geographic range, including the United States, Puerto Rico, the United Kingdom, Germany, Pakistan, China, and Iraq, reflecting diverse cultural, linguistic, and sociopolitical contexts.

**Figure 12:**
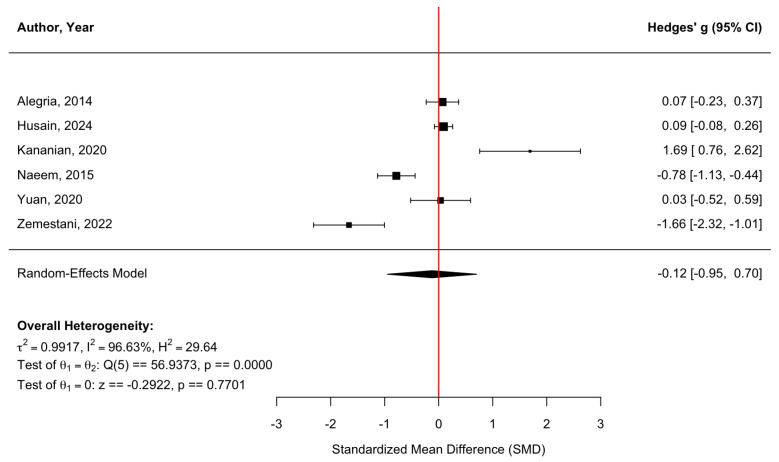
Forest plot of the effect of culturally adapted CBT on quality of life across diverse populations. Note. The following quality-of-life scales were used: **World Health Organization Disability Assessment Schedule 2.0 (WHODAS 2.0)** (Alegría et al., 2014), **EuroQol 5-Dimension 3-Level Visual Analog Scale (EQ-5D-3L VAS)** (Husain et al., 2024), **World Health Organization Quality of Life—Brief Version (WHOQOL-BREF)** (Kananian et al., 2020; Yuan et al., 2020; Zemestani et al., 2022), and **Bangladesh Disability Questionnaire (BDQ)** (Naeem et al., 2015).

**Figure 13:**
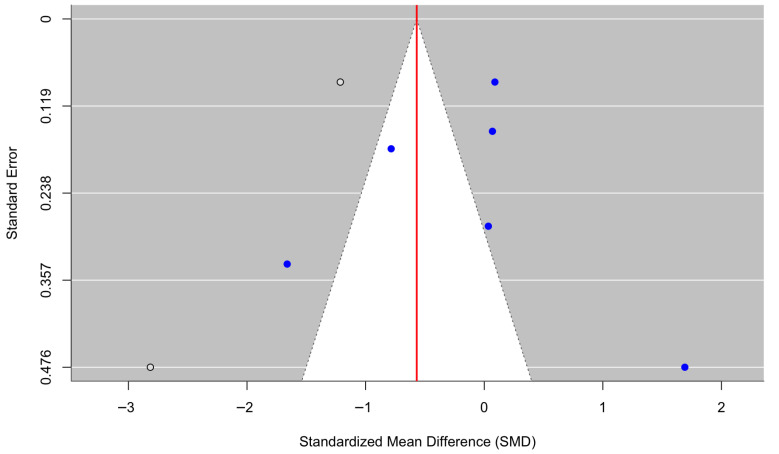
Adjusted funnel plot of the effect of culturally adapted CBT on quality of life across diverse populations using a random-effects model.

Figure 12 presents a forest plot of six randomized controlled trials evaluating quality-of-life outcomes. Overall, culturally adapted CBT did not produce a statistically significant improvement in quality of life relative to comparison conditions, with a pooled effect size of g = −0.12 (95% CI [−0.95, 0.70], z = −0.29, *p* = 0.770). Heterogeneity was substantial (τ^2^ = 0.99, τ = 1.00, I^2^ = 96.63%, H^2^ = 29.64), indicating wide variability across studies (Q(5) = 56.94, *p* < 0.0001). The high heterogeneity (I^2^ = 96.63%) limits the interpretability of pooled estimates and thus should be considered as descriptive summaries rather than definitive estimates of treatment benefit. The funnel plot (Figure 13) showed no evidence of significant publication bias. Egger’s regression test was nonsignificant (z = 0.68, *p* = 0.495), suggesting that small-study effects were unlikely. However, trim-and-fill analysis imputed two studies on the left side of the funnel, resulting in an adjusted pooled effect of g = −0.57 (95% CI [−1.46, 0.32], *p* = 0.211) based on an expanded model (k = 8). Although the adjusted estimate indicated a somewhat larger negative effect, neither the original nor the adjusted pooled estimates provided evidence of a statistically significant benefit for quality-of-life outcomes.

### 3.7. Emotional Regulation

For studies measuring emotional regulation, a total of 120 individuals were enrolled, with five withdrawals, resulting in a final sample of 116 participants. Two studies (*n* = 2) implemented intent-to-treat (ITT) analyses to account for missing data (Kananian et al., 2020; Zemestani et al., 2022). Two studies (*n* = 2) did not report any information on attrition (Hinton et al., 2009, 2011). After accounting for losses to follow-up, 60 participants in the intervention groups and 60 participants in the control groups were included in the analyzed sample across the four studies reporting post-treatment outcomes. The sample comprised 86 female and 34 male participants at enrollment, with two studies (*n* = 2) focusing exclusively on women (e.g., postnatal or refugee populations) (Hinton et al., 2011; Zemestani et al., 2022). The average mean age across studies was 38.5 years (SD = 6.1), calculated from four studies (*n* = 4) reporting numerical age data (Hinton et al., 2009, 2011; Kananian et al., 2020; Zemestani et al., 2022). Participants across the included studies primarily represented refugee, immigrant, and minority populations from Middle Eastern, Southeast Asian, and Caribbean–Latina backgrounds. These included Caribbean–Latina women of Dominican and Puerto Rican origin, Cambodian refugees who were Buddhist, Khmer-speaking survivors of the Khmer Rouge genocide, Afghan refugees from Pashtun, Tajik, and Hazara ethnic groups, and Iraqi Kurdish women exposed to war-related trauma. Studies were conducted across the United States, Germany, and Iraq, reflecting culturally diverse and sociopolitically complex settings relevant to trauma-exposed and migrant communities.

**Figure 14:**
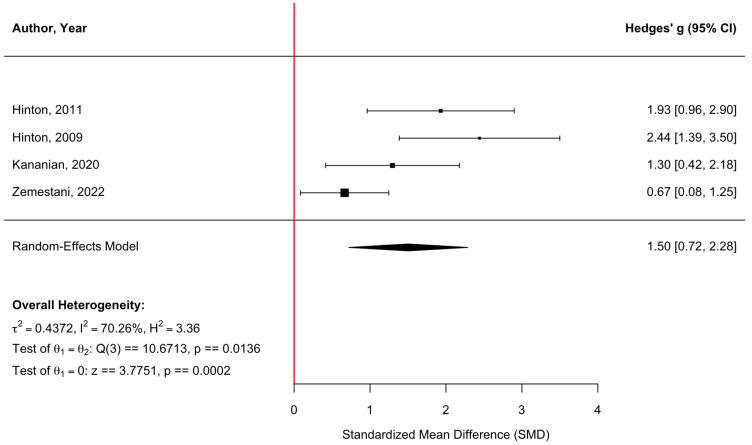
Forest plot of the effect of culturally adapted CBT on emotional regulation across diverse populations. Note. The following emotional regulation scales were used: **Emotion Regulation Scale (ERS)** (Hinton et al., 2009, 2011; Kananian et al., 2020) and **Difficulties in Emotion Regulation Scale (DERS)** (Zemestani et al., 2022).

**Figure 15:**
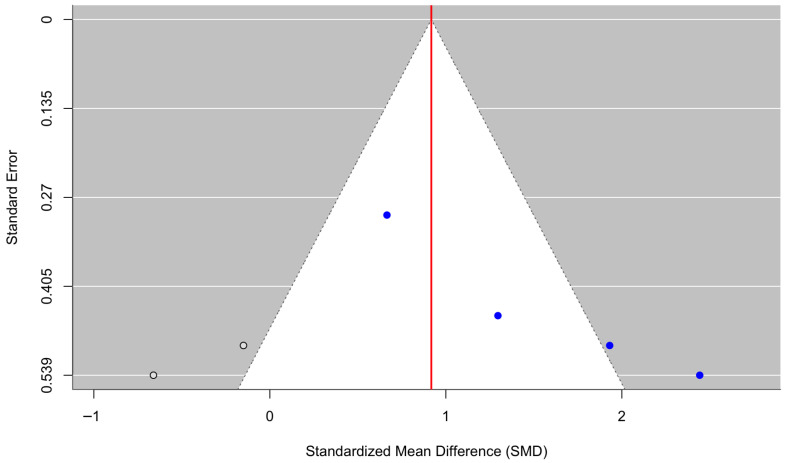
Adjusted funnel plot of the effect of culturally adapted CBT on emotional regulation across diverse populations using a random-effects model.

Figure 14 displays a forest plot of four randomized controlled trials assessing emotional regulation outcomes. Culturally adapted CBT produced a significant improvement in emotional regulation, with a pooled effect size of g = 1.50 (95% CI [0.72, 2.28], z = 3.78, *p* = 0.0002). Heterogeneity was moderate to substantial (τ^2^ = 0.44, τ = 0.66, I^2^ = 70.26%, H^2^ = 3.36), indicating variability across studies (Q(3) = 10.67, *p* = 0.0136). The funnel plot (Figure 15) suggested asymmetry, and Egger’s regression test was statistically significant (z = 3.15, *p* = 0.0016), indicating potential small-study effects. Trim-and-fill analysis imputed two studies on the left side, yielding an adjusted pooled effect size of g = 0.92 (95% CI [−0.01, 1.84], *p* = 0.052) based on the expanded model (k = 6). Although the adjusted estimate was attenuated and no longer statistically significant at the conventional α = 0.05 threshold, these findings should be interpreted cautiously given the small number of included studies and sensitivity to small-study effects and heterogeneity.

## 4. Discussion

This meta-analysis was the first to address a critical gap in the literature surrounding the effectiveness of culturally adapted cognitive behavioral therapy (CaCBT) across a broad range of mental health outcomes. The strongest and most consistent effects were observed for anxiety and depression, where culturally adapted interventions led to notable reductions in symptom severity across diverse populations. Findings for PTSD were more variable, although several studies showed meaningful symptom improvement; the overall pattern suggested that CaCBT may be less consistently effective for PTSD compared to mood and anxiety outcomes. Beyond the primary outcomes, CaCBT also demonstrated benefits for stress and emotional distress, somatic symptoms, and emotional regulation. However, improvements in quality of life were less consistent across studies. Although several nonsignificant outcomes showed point estimates in the direction of benefit for culturally adapted CBT, these patterns should be interpreted cautiously and do not constitute evidence of a statistical trend. Notably, across several nonsignificant outcomes, effect estimates were directionally consistent with benefit for CaCBT, suggesting that observed effects were not randomly oriented, despite overlapping confidence intervals. Findings derived from smaller subsets of studies should be interpreted cautiously, as limited study counts reduce precision and increase sensitivity to individual trials. Together, these findings highlight CaCBT’s value in strengthening culturally responsive care, particularly for emotional and stress-related outcomes, while underscoring the need for further research in trauma-focused and functional domains.

Given the magnitude of between-study heterogeneity observed (I^2^ frequently exceeding 75–90%), pooled effect estimates should be interpreted as descriptive summaries of highly variable effects across studies and contexts, rather than as reflecting a single underlying treatment effect.

The high heterogeneity observed across several outcomes reflects substantial variation in terms of populations, cultural adaptations, intervention formats, and outcome measures. As such, pooled estimates are presented as descriptive summaries of overall patterns rather than as stable or generalizable treatment effects.

PTSD outcomes were analyzed separately as a diagnostic trauma-related construct, whereas stress and emotional distress outcomes reflected non-diagnostic indicators of general psychological distress. Given the substantial heterogeneity observed across studies, pooled estimates were intended to summarize overall patterns rather than estimate a single uniform treatment effect. Accordingly, findings for these outcomes should be interpreted descriptively rather than definitively, particularly where confidence intervals overlapped the null.

Culturally adaptive cognitive behavioral therapy (CaCBT) is gaining importance as mental health care increasingly recognizes the influence of race, culture, and religion on treatment outcomes. The shift reflects broader social and health changes driven by globalization, which have prompted greater attention to psychosocial interventions, particularly in developing regions such as Asia (Naeem et al., 2019). By adapting CBT to align with cultural values and practices, therapists can deliver care that is more relevant and effective for diverse populations. These adaptations focus on developing cultural awareness, knowledge, and skills while preserving the core principles of CBT (Naeem, 2011).

CaCBT offers significant advantages in improving mental health outcomes for ethnocultural groups that may not benefit from standard CBT. A major benefit of CaCBT is the capacity to increase engagement, treatment retention, and symptom reduction by aligning therapeutic elements with clients’ cultural values (Griner & Smith, 2006). Furthermore, research shows that adapting CBT to account for clients’ beliefs, stigma, and communication of mental illness supports a better therapeutic experience among minority populations who are often less likely to receive adequate psychotherapeutic care (Hwang, 2006).

Meta-analytic evidence supports the superior efficacy of CA-CBT, with studies demonstrating that comprehensive adaptations yield the best outcomes. For instance, van Loon et al. (2013) systematically evaluated CA-CBT’s effectiveness for ethnic minority outpatients and found it was not only effective but also superior to standard care in treating symptoms of depression and anxiety. Anik et al. (2021) conducted a meta-analysis specifically for adults of African and Caribbean descent, finding that CA-CBT produced substantial reductions in depression and anxiety symptoms and that more comprehensive adaptations were correlated with larger effect sizes. Similarly, Escobar & Gorey (2018) reviewed culturally adapted group therapies for Latino immigrants with depression, determining that key effective components included delivery in Spanish and the integration of cultural values. Beyond mood and anxiety disorders, evidence extends to other common mental health conditions: CA-CBT reduces depression among women experiencing infertility in South Punjab, Pakistan (Sultana et al., 2024), supports recovery in postnatal depression among British South Asian women (Husain et al., 2024), and decreases non-suicidal self-injury in a Pakistani sample (Kumar et al., 2024). It has also shown benefits for serious mental illness and trauma, including psychosis in low- and middle-income settings (Naeem et al., 2015), PTSD among Latina patients with treatment-resistant presentations (Hinton et al., 2011), PTSD in refugee groups (Menon et al., 2024), and panic disorder with agoraphobia (Subhas et al., 2021).

Recent evidence continues to support the effectiveness of culturally adapted CBT for diverse populations. For example, a meta-analysis by Silveus et al. (2023) synthesized between-group trials and found that CaCBT yielded moderate to large effect sizes, reducing both anxiety and depression symptoms compared to active control conditions. These results build on earlier evidence from Griner and Smith (2006), whose meta-analysis found that culturally adapted interventions demonstrated substantially greater effects when tailored to a specific cultural group. Evidence also extended this to digital and self-help versions of CaCBT, resulting in a strong positive impact, particularly through the inclusion of adapted interventions, such as using clients’ native language (Shehadeh et al., 2016). These findings directly support the results of our meta-analysis, which similarly found that CaCBT is effective in reducing symptoms of anxiety, depression, and PTSD, particularly when adaptations are made thoughtfully and tailored to specific cultural contexts. Our work extends this evidence by demonstrating that CaCBT is a valuable intervention.

## 5. Limitations

A key limitation of this meta-analysis is the relatively small number of included studies, primarily because articles that otherwise met eligibility criteria lacked complete statistical reporting. Out of 37 eligible studies, this meta-analysis reports on the outcomes of 16 articles due to the missing data and unsuccessful attempts to reach authors for 21 studies. This limited dataset restricts the strength and precision of pooled estimates and increases uncertainty and reduces the precision of the pooled estimates, particularly due to the possibility of inflated effect sizes in studies with a smaller sample. Some studies also showed substantial variability in control conditions. Many trials used treatment-as-usual (TAU) or other control groups that differed in structure, content, or level of engagement per intervention. This inconsistency complicated direct comparisons across studies and influenced the magnitude of observed effects. Additionally, while the review focused on outcomes related to anxiety, depression, and PTSD, other relevant mental health domains such as overall psychological well-being, stress, and broader functional outcomes were not consistently assessed, leaving potentially important therapeutic effects unexamined. Similarly, different studies looked at mental health outcomes using varied scales and relied on self-report measures. It is important to note that the reliance on self-reporting for mental health may yield different outcomes in comparison to if a clinician had confirmed a diagnosis during the process.

Additionally, the assessment of publication bias for emotional regulation outcomes should be interpreted cautiously. Funnel plot-based methods and trim-and-fill procedures are underpowered and unreliable when the number of included studies is small. Given the limited number of studies contributing to these outcomes, the presence or absence of funnel plot asymmetry cannot be taken as definitive evidence for or against publication bias.

Another significant limitation is the demographic composition of the included samples, which were predominantly from South Asia. This raises questions about the generalizability of CaCBT across different cultural or clinical contexts, where adaptation requirements and outcomes may vary. Finally, the exclusive reliance on quantitative data limited our ability to evaluate the qualitative implementation of CaCBT, such as patient satisfaction and cultural resonance. Qualitative and mixed-method research would be valuable in capturing these experiential factors.

Finally, although outcomes were grouped based on conceptual alignment, pooling across non-diagnostic distress measures may still obscure meaningful differences among stress-related constructs, further reinforcing the need to interpret pooled estimates as descriptive rather than definitive evidence.

## 6. Future Directions

Future studies should examine a broader range of mental health outcomes, including trauma-related symptoms, adjustment disorders, and well-being indicators that may also benefit from culturally adapted interventions. There is also a need for qualitative and mixed-methods research to complement quantitative findings, providing insight into the lived experience, acceptability, feasibility, efficacy, and nuanced ways in which adaptation influences engagement and therapeutic change. This aligns with emerging research that leaves room for dedicated synthesis integrating patient perspectives, implementation barriers, and clinician experience.

Evidence supports the effectiveness of CaCBT in improving depression and anxiety outcomes across diverse populations. However, most trials apply multiple cultural adaptations concurrently, limiting the ability to determine which specific components (e.g., language matching, cultural metaphors, family involvement) independently drive treatment effects. Consequently, while CaCBT appears beneficial as an integrated approach, the intervention components underlying its effectiveness remain unclear.

Additionally, future studies should explore culturally adaptive versions of other therapeutic modalities beyond CBT (e.g., ACT, DBT) to determine whether cultural tailoring enhances their effectiveness across diverse clinical needs. This would help clarify whether cultural adaptation is beneficial at the level of therapeutic principle more broadly and deepen our understanding of how culturally informed psychotherapy can be optimized and generalized across different treatment models while maintaining its resonance for diverse populations.

Although future research is needed, this meta-analysis suggests the potential benefit of CaCBT for selected mental health outcomes among individuals of non-Western cultural backgrounds, particularly in individual therapy formats. Clinical implications of this work include culturally revising how CBT may be delivered, and future work should also consider effectiveness in comparison to group CBT, whether family therapy or otherwise, and explore the role of guided self-help versus therapist-administered therapy.

Future research should also explore why CaCBT might be less effective for PTSD and quality-of-life outcomes. There exists significant data on depression and anxiety outcomes for CaCBT, with this meta-analysis providing a foundation for insight into these variables; however, certain outcomes, such as PTSD and quality of life, are less explored. Future studies should aim to integrate scales for these variables to help provide insight into how effective CaCBT can be for quality of life in the physical and mental health domains and for PTSD, especially for individuals who have experienced trauma.

## 7. Conclusions

This meta-analysis indicates potential benefits of culturally adapted CBT for anxiety, depression, and PTSD symptoms compared to TAU within a heterogeneous evidence base. While CBT is a well-established evidence-based intervention, much of the existing literature is rooted in Western models that do not fully account for cultural beliefs, values, and systematic barriers shaping treatment responses among ethnic minorities and non-Western populations. By tailoring therapeutic content and delivery to align with clients’ cultural contexts, CaCBT appears to enhance both engagement and clinical outcomes. These results are consistent with prior meta-analyses, indicating that culturally adapted interventions have substantially contributed to treatment effectiveness.

However, the relatively small number of eligible studies, variability in control conditions, and the predominance of South Asian samples reduce the generalizability of the results across broader cultural and clinical contexts. Additionally, the lack of qualitative data limits insight into client experiences and therapeutic processes that are likely to add to the effectiveness of CaCBT but are still underexplored. Future research should include more diverse populations, examine broader outcomes, and employ qualitative methods to understand how cultural adaptation shapes engagement and therapeutic change. Assessing these interventions will clarify whether they improve effectiveness across different therapeutic models.

## Figures and Tables

**Table 1:** Study characteristics.

Author	Country	Intervention Name	Study Design	Primary Outcomes	Mean Age (SD); Age Range	Intervention Description	List of Groups	Measure for Each Primary Outcome
Alegría et al. (2014)	United States (Boston) and Puerto Rico (San Juan)	ECLA	Multisite RCT	Depression severity; functional impairment	45 (mean derived), SD n/r; 18–65+	A six- to eight-session CA-CBT program for low-income Latino adults offered by phone or in person, combining basic CBT skills with help navigating practical barriers to depression care.	(1) ECLA-F—Int (2) Usual Care—Con (3) Telephone CBT (not analyzed)	PHQ-9; HSCL-20 (not analyzed); WHO-DAS 2.0
Dwight-Johnson et al. (2011)	United States (Rural Washington State)	T-CBT	RCT	Depression severity	Telephone CBT 41.17 (9.69); EUC 38.54 (10.27); adults ≥18 (no upper limit)	Eight-session CaCBT delivered by phone by bilingual community therapists to rural Latino primary care patients using a Spanish-language workbook with behavioral activation and guided homework.	(1) Telephone CBT—Int (2) Enhanced Usual Care—Con	PHQ-9; SCL-20 (not analyzed); Satisfaction Scale
Eskici et al. (2021)	Turkey	CA-CBT	RCT	PTSD; anxious–depressive distress	CA-CBT 33.6 (8.5), 24–55; TAU 36.8 (8.1), 28–50	Seven-session group CaCBT for Syrian refugee women, delivered weekly by Arabic-speaking facilitators and adapted with Syrian idioms, somatic techniques, emotion regulation, mindfulness, and culturally meaningful imagery.	(1) CA-CBT—Int (2) TAU—Con	HTQ; HSCL-25 (depression and anxiety)
Hinton et al. (2005)	United States (Lowell, MA)	CA-CBT	Cross-over RCT	PTSD; panic attacks; anxiety; depression	Immediate treatment 50.90 (6.11); delayed treatment 52.70 (7.43)	Twelve-session CaCBT for Cambodian refugees targeting neck-focused and orthostatic panic through mindfulness, breathing, stretching, interoceptive/trauma-focused exposure, and cognitive restructuring.	(1) Immediate Treatment—Int (2) Delayed Treatment—Con	CAPS; ASI; SCL-90-R (anxiety and depression); N-PASS (not analyzed); O-FSS (not analyzed); O-PASS (not analyzed; N-FSS (not analysed)
Hinton et al. (2009)	United States (Lowell, MA)	CA-CBT	Mechanism-focused RCT	PTSD; orthostatic panic; emotion regulation	Immediate treatment 49.92 (9.23); delayed treatment 49.08 (7.56)	Twelve-session CaCBT for Cambodian refugees targeting orthostatic panic and PTSD through emotion-regulation training, somatic techniques, mindfulness, stretching, breathing, and interoceptive exposure.	(1) Immediate Treatment—Int (2) Delayed Treatment—Con	CAPS; Emotion Regulation Scale; O-PASS; O-FSS (not analyzed); O-CCSS (not analyzed); Orthostatic BP Response (not analyzed)
Hinton et al. (2011)	United States (Urban Massachusetts)	CA-CBT	RCT	PTSD; anxiety; nervios; ataque de nervios; emotion regulation	CA-CBT 47.6 (8.2); AMR 51.4 (5.9)	Fourteen-session group CaCBT for Caribbean-Latina women with treatment-resistant PTSD, using somatic focus, emotion-regulation, culturally adapted imagery (e.g., Sagrado Corazón), interoceptive exposure, stretching, and cognitive restructuring of culture-specific expressions.	(1) CA-CBT—Int (2) AMR—Con	PTSD Checklist; SCL-90-R Anxiety; Nervios Scale (not analyzed); Emotion Regulation Scale
Husain et al. (2024)	United Kingdom (Manchester, Yorkshire, East Midlands, London, Glasgow)	PHP	Multicenter RCT	Postnatal depression	PHP 31.3 (5.2); TAU 31.4 (5.2); 16+	Twelve-session group CaCBT-based PHP for British South Asian women with postnatal depression, delivered by non-specialist facilitators using culturally adapted CBT, mood education, relaxation, and support for identity, family stress, and social isolation.	(1) PHP—Int (2) TAU—Con	HDRS (not analyzed); PHQ-9; GAD-7; Social Functioning Scale (not analyzed); EQ-5D-3L; Parenting Sense of Competence Scale (not analyzed)
Hwang et al. (2015)	United States (San Francisco and Los Angeles)	CA-CBT for Chinese Americans	RCT	Depression; retention	CA-CBT 44.8 (10.7); CBT 45.7 (12.6); 18–65	Twelve-session individual CaCBT for Chinese American adults with major depression, incorporating cultural metaphors, stigma reduction, somatic framing, goal setting, and culturally congruent communication alongside standard CBT skills.	(1) CA-CBT—Int (2) Standard CBT—Con	HDRS; SCID (not analyzed)
Jalal et al. (2020)	South Africa	CA-CBT	RCT (CA-CBT vs AMR)	PTSD; anxiety; depression	CA-CBT 28.2 (SD n/r); AMR 28.2 (SD n/r); age range n/r	Fourteen-session individual CaCBT for Sepedi-speaking traumatized South Africans, emphasizing somatic-focused exposure, yoga-like stretching, meditation, emotion-regulation, and culturally grounded explanations for symptoms.	(1) CA-CBT—Int (2) AMR—Con	PCL-C; HARS; BDI-II; Sepedi SSA (somatic symptoms and cultural syndromes)
Kananian et al. (2020)	Germany (Frankfurt)	CA-CBT+	RCT	General psychopathology; PTSD; depression; somatic symptoms; quality of life; emotion regulation	CA-CBT+ 21.0 (3.4), 18–29; qaitlist 22.8 (3.3), 18–29	Twelve-session group CaCBT+ for Farsi-speaking Afghan male refugees, combining culturally adapted CBT with stretching, meditation, idioms of distress, and added problem-solving training to address post-migration stressors.	(1) CA-CBT+—Int (2) Waitlist—Con	YBOCS; BDI; BAI
Karadere et al. (2024)	Turkey	CA-CBT (Group CBT with ERP)	RCT	OCD; depression; anxiety	CBGT 28.60 (SD n/r); control 29.35 (SD n/r); 18–65	Fourteen-session group CaCBT for Turkish-speaking adults with OCD, culturally adapted with Turkish idioms, religious/cognitive themes, and ERP-based CBT tools to reduce obsessions, compulsions, anxiety, and depression.	(1) CBGT—Int (2) Befriending Therapy—Con	HADS-D; HADS-A; Bradford Somatic Inventory; Brief Disability Questionnaire
Naeem et al. (2015)	Pakistan (Lahore)	CA-CBT (brief)	Assessor-blind RCT	Depression; anxiety; somatic symptoms; disability	CA-CBT 30.0 (11.4); TAU 33.4 (10.7); age range 18–64	Six-session CaCBT for depression in Pakistan, delivered with a family-supported format using psychoeducation, behavioral activation, culturally adapted thought-records, Urdu CBT terms, and locally relevant stories/examples.	(1) Brief CA-CBT + TAU—Int (2) Treatment As Usual (TAU)—Con	HADS-D; HADS-A; Bradford Somatic Inventory; Brief Disability Questionnaire
Shaw et al. (2019)	Malaysia (Kuala Lumpur)	CA-CBT (somatic-focu sed)	RCT + Non-randomized arm	Emotional distress; anxiety; depression; PTSD; social support	G1 32.35 (11.60); G2 30.78 (4.55); G3 40.10 (14.28); age range n/r	Eight-session group CaCBT for Afghan refugee women in Malaysia, using somatic-focused emotion-regulation skills, stretching, breathing, mindfulness, visualization, cognitive restructuring, and interoceptive exposure, delivered by a trained community facilitator.	(1) Initial Treatment—Int (2) Waitlist Control—Con (3) Non-randomized Treatment Group—Additional Int	RHS-15 (not analyzed); HSCL-25 (anxiety, depression); HTQ (PTSD); MOS Social Support (not analyzed)
Simoni et al. (2013)	United States (El Paso, Texas)	CBT-AD	Preliminary RCT	Depression; ART adherence; viral load; CD4	CBT-AD 47.3 (10.7); TAU 44.8 (10.7); 24–63	More than twelve-sessions CaCBT-AD for HIV-positive Latinos integrating adherence counseling, behavioral activation, cognitive restructuring, problem solving, relaxation, and bilingual delivery alongside an electronic pillbox reminder system.	(1) CBT-AD—Int (2) TAU (enhanced)—Con	BDI-IA; MADRS (not analyzed); EDM (electronic pillbox) (not analyzed); VAS adherence (not analyzed); CD4 (not analyzed); Viral load (not analyzed)
Yuan et al. (2020)	China (Rural Sichuan)	CA-CBT (lay-delivered)	Pilot RCT	Depression severity; anxiety; social relationships	CA-CBT 70.5 (5.6), 64–90; CAU 70.5 (5.6), 64–90	Eight-session CaCBT for older adults in rural China delivered by trained lay health workers, using culturally adapted language, proverbs, somatic check-ins, and tailored behavioral activation to fit education level, daily routines, and local dialect.	(1) CA-CBT—Int (2) CAU—Con	GDS; SAS; WHOQOL-BR EF (domain 3)
Zemestani et al. (2022)	Iraq (Kurdistan)	TF-CBT	RCT	PTSD; depression; anxiety; stress	TF-CBT 33.45 (5.46), 18–50; WLC 32.37 (5.27), 18–50	Twelve-session culturally adapted trauma-focused CBT for Iraqi Kurdish women with war-related PTSD, integrating local language, proverbs, idioms of distress, culturally relevant emotion-regulation practices, trauma narration, cognitive restructuring, and in vivo exposure.	(1) TF-CBT—Int (2) WLC—Con	PCL-5; DASS-21 (anxiety, stress, and depression); DERS; WHOQOL-BR EF

Note. AMR: applied muscle relaxation; ASI: Anxiety Sensitivity Index; BAI: Beck Anxiety Inventory; BDI-II: Beck Depression Inventory–II; CA-CBT: culturally adapted cognitive behavioral therapy; CA-CBT+: culturally adapted cognitive behavioral therapy + problem solving; CAU: care as usual; CAPS: clinician-administered PTSD scale; CBT: cognitive behavioral therapy; CBT-AD: cognitive behavioral therapy for adherence and depression; CBGT: cognitive behavioral group therapy; CES-D: center for epidemiologic studies depression scale; Con: control; DASS-21: Depression Anxiety Stress Scale–21; DERS: Difficulties in Emotion Regulation Scale; ECLA: engagement and counseling for Latinos; EDM: electronic drug monitor; EUC: enhanced usual care; EQ-5D-3L: EuroQol 5-dimension 3-level scale; ERS: Emotion Regulation Scale; F2F: face-to-face; FMAP: Formative Method for Adapting Psychotherapy; GAD-7: Generalized Anxiety Disorder–7; GDS: Geriatric Depression Scale; GHQ-28: General Health Questionnaire–28; HADS-A: Hospital Anxiety and Depression Scale—Anxiety Subscale; HADS-D: Hospital Anxiety and Depression Scale—Depression Subscale; HARS: Hamilton Anxiety Rating Scale; HDRS: Hamilton Depression Rating Scale; HPASS: Headache Panic Attack Severity Scale; HSCL-20: Hopkins Symptom Checklist–20; HSCL-25: Hopkins Symptom Checklist–25; HTQ: Harvard Trauma Questionnaire; Int: Intervention; MADRS: Montgomery–Åsberg Depression Rating Scale; MOS-SS: Medical Outcomes Study–Social Support; N-FSS: Neck Fear and Somatic Symptoms Scale; N-PASS: Neck Pain and Autonomic Symptoms Scale; O-CCSS: Orthostatic Catastrophic Cognition Severity Scale; O-FSS: Orthostatic Fear and Somatic Symptoms Scale; O-PASS: Orthostatic Panic Attack Severity Scale; OCD: obsessive–compulsive disorder; PAMF: Psychotherapy Adaptation and Modification Framework; PCL-C: PTSD Checklist–Civilian Version; PCL-5: PTSD Checklist for DSM-5; PHP: Positive Health Program; PHQ-9: Patient Health Questionnaire–9; PTSD: post-traumatic stress disorder; RCT: randomized controlled trial; RHS-15: Refugee Health Screener–15; SAS: Self-Rating Anxiety Scale; SCL-20: Hopkins Symptom Checklist–20 Depression Items; SCL-25: Hopkins Symptom Checklist–25; SCID: Structured Clinical Interview for DSM Disorders; SD: standard deviation; SSA: Sepedi Symptom and Syndrome Addendum; SSS-8: Somatic Symptom Scale–8; TAU: treatment as usual; TCMIE: Trauma–Catastrophic–Metaphoric–Interoceptive Escalation; TF-CBT: trauma-focused cognitive behavioral therapy; WLC: waitlist control; WHO-DAS 2.0: World Health Organization Disability Assessment Schedule 2.0; WHOQOL-BREF: World Health Organization Quality of Life–Brief; YBOCS (Y-BOCS): Yale-Brown Obsessive–Compulsive Scale.

**Table 2:** Risk of bias estimates in individual trials (*n* = 16).

Study	Sequence Generation	Allocation Concealment	Blinding (Participants /Personnel)	Blinding of Outcome Assessors	Incomplete Outcome Data	Selective Outcome Reporting	Other Bias
Alegría et al. (2014)	Low Risk	Unclear	High Risk	Unclear	Low Risk	Low Risk	Unclear
Dwight-Johnson et al. (2011)	Low Risk	Low Risk	High Risk	Unclear	Low Risk	Low Risk	Unclear
Eskici et al. (2021)	Low Risk	Low Risk	High Risk	Low risk	High Risk	Low Risk	Unclear
Hinton et al. (2005)	Low Risk	Unclear	High Risk	Low Risk	Low Risk	Low Risk	Unclear
Hinton et al. (2009)	Low Risk	Unclear	High Risk	Low Risk	Low Risk	Low Risk	Unclear
Hinton et al. (2011)	Unclear	Unclear	High risk	High Risk	Low risk	Low risk	High Risk
Husain et al. (2024)	Low Risk	Low Risk	High Risk	Low Risk	Low Risk	Low Risk	Unclear
Hwang et al. (2015)	Low Risk	Low Risk	Low Risk	Unclear	Low Risk	Low Risk	Low Risk
Jalal et al. (2020)	Low Risk	Low Risk	Low Risk	Unclear	Low Risk	Low Risk	Unclear
Kananian et al. (2020)	Low Risk	Low Risk	Unclear	Unclear	Low Risk	Low Risk	Low Risk
Karadere et al. (2024)	Low Risk	High Risk	High Risk	High risk	Low Risk	Low Risk	Unclear
Naeem et al. (2015)	Unclear	Low Risk	Low risk	Unclear	Low Risk	Low Risk	Low Risk
Shaw et al. (2019)	Low Risk	Unclear	High Risk	High risk	Low Risk	Low Risk	High Risk
Simoni et al. (2013)	Low Risk	Low Risk	High Risk	High Risk	Unclear	Low Risk	Low Risk
Yuan et al. (2020)	Low Risk	Unclear	High Risk	Low Risk	Low Risk	Low Risk	High Risk
Zemestani et al. (2022)	Low risk	Unclear	High Risk	High Risk	Low Risk	Low Risk	Low Risk

## Data Availability

No new data were created or analyzed in this study. Data sharing is not applicable to this article.

## Supplementary Materials

The following supporting information can be downloaded at https://www.mdpi.com/article/10.3390/bs16030356/s1: File S1: PRIMSA Checklist.
